# The Changing Discourse of Healthism: A Contextual Analysis

**DOI:** 10.1111/1467-9566.70073

**Published:** 2025-08-02

**Authors:** David Armstrong

**Affiliations:** ^1^ Department of Population Health Sciences King's College London London UK

**Keywords:** behaviour, discourse, healthism, medicalisation

## Abstract

This article uses bibliometric and thematic analyses to explore the origins and influence of Crawford's 1980 paper on healthism and the medicalisation of everyday life. The construct of healthism was built on some important concepts such as medical dominance/power, medicalisation, alternative medicines, lifestyles and health behaviour that had only first emerged during the previous decade. In the new millennium, however, healthism has become more associated with new ideas such as appearance and neoliberalism and with wider debates about self‐responsibility. This shift in context was also found in the patterning of citations to the paper. After an initial slow accumulation of citations, the number grew rapidly from about 2005. With increasing involvement of patients in their own care management (especially for long‐term conditions) and the promotion of more shared decision‐making in clinical encounters, the role of self‐responsibility in the healthism literature increasingly reflects the emergence of a ‘subjectified’ individual.

## 1 Introduction

What makes a publication influential? Why has Crawford’s 1980 paper on healthism and the medicalisation of everyday life been described as ‘seminal’ (Markula‐Denison and Pringle 2007), ‘influential’ (Dworkin and Wachs 2009) and ‘classic’ (Gard 2011; Lewis and Potter 2013)? In academia, the usual hallmark of influence lies in the number of citations a publication accumulates. Yet, many factors have been reported as influencing citation rates, not only the quality of the paper or the novelty and interest of the subject (Tahamtan et al. 2016). Any publication exists as a node in a wider matrix of other publications that come before it and after it. The significance of subsequent citations to a paper may therefore range from channelling interest in previous publications to the use of a concept in a new or different framework. In short, it is both the content and context of citations, as well as their number, that might signify influence.

To identify the patterning of citations to Crawford's 1980 paper, this article used Google Scholar. Although citation indices such as Scopus and Web of Science have been frequently used to study scientific disciplines, they have limited coverage of some journals and some subject areas. Google Scholar has therefore emerged as an alternative resource, as it covers a wider field of publications including books, an important resource in social science (Martín‐Martín et al. 2018). Although Google Scholar lacks the quality control of Scopus and Web of Science (Aguillo 2012; Jacsó 2008), it is still useful for recognising broad trends, and its results have been shown to be a reliable means of identifying highly cited documents (Martin‐Martin et al. 2017, 2016). All citations in Google Scholar to Crawford's paper were therefore collated, and a count by year was carried out. This enabled a rough assessment of the influence of the paper over time.

Publications citing healthism not only promote the original paper but might also magnify its potential influence and reach if they themselves are frequently cited. An arbitrary cut‐off of more than 200 citations was used to identify publications that might have played a bigger role in the dissemination of Crawford's paper against those that had a smaller impact. This process identified 116 publications (two non‐English language citations were excluded). Most of these publications were then identified through library collections and online databases (particularly the Internet Archive for books), and their content was searched for reference to healthism and Crawford's index paper. It was quickly apparent that the meaning of the word and concept of healthism had not changed over time but had been deployed in support of a number of different themes and arguments. These ‘contexts’ were then grouped thematically.

This approach has two important limitations. First, by concentrating on ‘highly cited’ citations, it may have missed publications that had fewer citations yet collectively made important contributions to understanding the post‐1980 history of healthism. Second, given the coverage of Google Scholar, it is restricted to ‘academic’ publications and ignores the extent to which healthism may have entered popular discourse. Turrini, for example, claimed that the concept of healthism had been relatively neglected by social science but circulated widely outside of academia and in different countries; indeed, the term had been translated into several languages as it had become ‘a common everyday word’ (Turrini 2015). Moreover, in this ‘lay’ discourse, healthism was often given a positive spin, unlike the academic publications reviewed here that echoed Crawford's original critical perspective. In examining the ‘genealogy of healthism’, Turrini noted its affinity with constructs such as risk factors and lifestyle, themes that also emerged from the highly cited papers analysed here in the academic literature. Turrini's contribution is further discussed in the concluding section of this article.

Finally, the references that Crawford himself used in his 1980 paper were examined, as his paper could simply have been a relay point in the influence of a predecessor publication or publications. Once more using Google Scholar, a database of the number of citations that each of Crawford's references has subsequently achieved was created. There were 73 references in the original paper, and all but five were drawn from the 1970s. Again, highly cited references were identified for further study, though Crawford's own analysis provides the main framework for their interpretation.

## 2 Citation Counts

**FIGURE 1:**
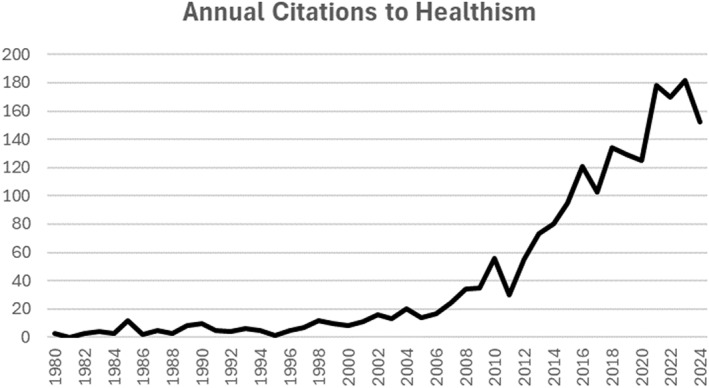
Annual citations to healthism (from Google Scholar).

According to Google Scholar, Crawford's 1980 paper ‘Healthism and the medicalisation of everyday life’ (Crawford 1980) has over 2000 citations. The distribution of these citing papers over the 4 decades following its publication is shown in Figure 1.

For the first 20–25‐year period, the number of citations was fairly muted before rising steeply. In general, the time between publication and peak citation rate seems to vary by discipline. In an analysis of 25 years of annual citation counts for over a thousand articles published in APA journals, for example, peak citation occurred 4 years after publication, but those articles classified as high impact (with over 250 citations) maintained their high annual count over the 25‐year period (Walters 2011). Citations in areas such as chemistry have been reported as peaking after 2 years, whereas management papers grow for over 10 years (Finardi 2014). Similarly, sociology papers have later peaks and longer declines than natural science papers (Galiani and Gálvez 2017). Nevertheless, an initial quiescent period of about 25 years for Crawford's paper is unusual but not unknown—what in bibliometrics has been called a ‘sleeping beauty’ (Lachance and Larivière 2014).

**FIGURE 2:**
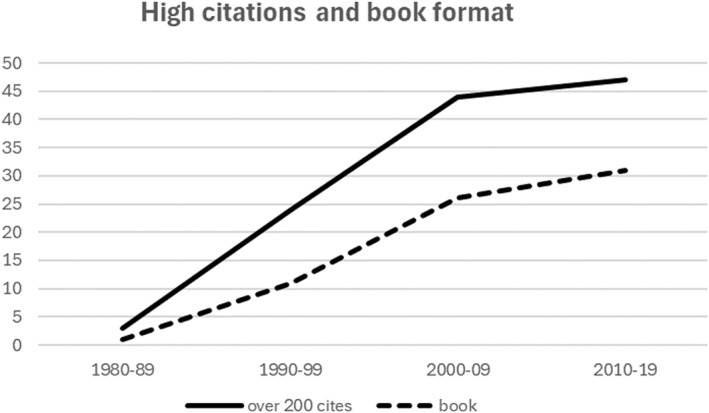
High citations by decade and whether book format.

Figure 2 shows the distribution of the ‘highly cited’ citations (i.e., with over 200 citations themselves) to Crawford's paper by decade. Figure 2 also shows the salience of books (as against journal articles) among subsequent highly cited publications that referenced Crawford's paper. Given that many more journal papers than books are published, the latter show a disproportionately high impact. Highly cited publications in the form of books reached about 65% between 2000 and 2009, though it has because levelled off to 40% in the most recent complete decade. Citations in the social sciences take time to accumulate, but books may have a longer life as a citation source, a factor supporting the use of Google Scholar for this analysis.

## 3 Before 1980: Recruiting Support

The role of references in an academic paper is to bring support for its arguments. As Latour noted, ‘The presence or the absence of references, quotations and footnotes is so much a sign that a document is serious or not that you can transform a fact into fiction or a fiction into fact just by adding or subtracting references’ (Latour 1987, 33). Therefore, when Crawford cited earlier publications, he was recruiting support for arguments that otherwise would have seemed less authoritative. In fact, he was quite explicit that he was building on earlier work: ‘This paper is a tentative assessment of some such efforts [to capture the concept of health] made in the late 1970s in the United States’ (p. 365).

Crawford acknowledged the term ‘healthism’, which summarised a new health consciousness, was borrowed from an earlier study by Zola, which had used the concept only in its title but claimed in the body of the paper that ‘Health itself became not merely the means to some larger end but the end in itself, no longer one of the essential pillars of the good life but the very definition of what is the good life’ (Zola 1977, 51). However, it was Zola's own earlier notion of medicalisation (Zola 1972) that offered the substantive underpinning for Crawford's study. According to Google Ngrams, which allows searching for the rate of use of specified words or phrases across over 5 million digitised books (Michel et al. 2011), the construct of medicalisation emerged in the 1970s (with Zola's paper) and then achieved rapid and widespread use. Crawford's paper on healthism was part of that enthusiasm for the concept, and medicalisation proved to be a frequent reference point for healthism in subsequent decades. Zola's elaboration of the term, together with Conrad and Schneider's further development (Conrad and Schneider 1980), formed two of Crawford's key references. Freidson's *Profession of Medicine* (over 10,000 citations) and Illich's *Medical Nemesis* (over 7000 citations; Freidson 1970; Illich 1976) offered further support for the way in which the medical profession defined what was to be counted as illness. These latter sources were also critical of medicine and the power it wielded, a theme that provided further context for Crawford's main argument.

During the 1970s, two ‘popular health movements’, holistic medicine and self‐care, had emerged that offered alternative ways of managing illness. According to Crawford, holistic medicine combined a sense of a unified body and mind, usually supported by alternative healers (the descriptor of ‘alternative medicine’ was also a construct of the 1970s). Crawford offered several references to the importance of holistic medicine, though none was subsequently extensively cited, perhaps reflecting that the primary audience was mainly lay rather than academic. Crawford observed that the self‐help and self‐care movement also claimed to offer challenges to conventional medicine by promoting self‐reliance rather than use of the health care system. His summary was supported by references that included several highly cited books, all drawn from the 1970s, indicating the then historical recency of the movement. Gartner and Riessman's book on self‐help of 1977, for example, has 649 citations, 216 from the 21st century, suggesting a continuing interest in the theme (Gartner and Riessman 1977).

There were then two pillars to Crawford's argument. On the one hand, the dominant medical profession was busy medicalising everyday problems; on the other hand, resistance to that dominance emerged in the form of alternative ways of healing. Yet, although both holism and self‐care offered challenges to medicine, they still located the problem (and solution) within the individual. According to Crawford, in endorsing reliance on individual action, both holistic health and self‐care models ignored the social constraints against making choices. In both cases, resistance to a dominant medicine still relied on the individual to fight back, not the collective; in both cases, their central premise was the concept of individual responsibility. And it was individual responsibility that underpinned his account of healthism and its critical evaluation.

**FIGURE 3:**
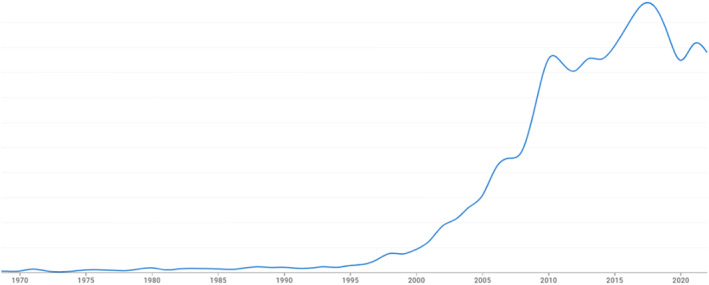
Rate of mentions of ‘self‐responsibility’ in Google Ngrams.

The ‘ideology of individual responsibility for health’ had been explored by Crawford in an earlier paper (Crawford 1977), but he claimed his 1980 paper examined this thesis in greater depth and breadth. Crawford still acknowledged that taking responsibility for health could have some positive impact; indeed, he cited a study (itself with over 2000 citations) showing that those who adopted this individual control model improved their own health (Belloc and Breslow 1972). However, it seemed too much to ask that everyone should—and, moreover, could—take responsibility for their own health. It was this theme that informed most of the subsequent citations to his paper. In fact, self‐responsibility was also a construct that only emerged in the 1970s, and although it was slow to grow, its rapid increase in the new millennium closely follows the rise in citations to Crawford's paper (see Figure 3).

Crawford endorsed Callahan's critique of the WHO's celebrated definition of health—a state of complete physical, mental and social well‐being—which cemented the position of health as a personal achievement (Callahan 1973). Indeed, health practices from other cultures not so dependent on individualism showed that radically different forms of health management were possible (Kleinman et al. 1978). However, the individualistic focus of 1970s Western societies was reinforced by the contemporary medical model that located health problems, as well as their immediate causes, within the individual patient's body. Crawford deployed Foucault's account (nearly 20,000 cites in its various editions) of the rise of modern pathological medicine at the end of the 18th century (Foucault 1973) to support this claim. Further, Crawford noted that this model of illness had failed to cure many chronic and degenerative diseases and had made a limited contribution to the decline of mortality from infectious diseases (Lalonde 1974; McKeown 1979).

At several points, Crawford quoted approvingly from a book by Ardell on ‘high‐level wellness’ that used lifestyle as a central theme (Ardell 1977). Although the word ‘lifestyle’ had been coined earlier in the century, Google Ngrams shows that it only became popular in the 1970s. Healthism was therefore another one of those various lifestyles that were being identified in the preceding decade. According to Crawford, health‐related lifestyles were highly influenced by medical propaganda that was ‘bombarding our culture with the message that health is the most important of values, offering its magic bullets as the key to longer and disease‐free lives’ (p. 382). On the other hand, resistance to that pressure produced lifestyles associated with the holistic and self‐help movements. To be sure, the new ‘potential sick role’ mandated an obligation to correct unhealthy habits (Knowles 1977), but the expectation that individuals had to look after their own health, irrespective of their capacity to do so, created social costs and distracted from the real basis of illness. Just as the language of caring or help obscured the unequal power relationships of a growing therapeutic state (Edelman 1974), so the language of ‘self‐care, individual responsibility and holism obscures the power relations underlying the social production of dis‐ease and discontent’ (p. 383).

Crawford's paper on healthism was, not unexpectedly, a product of its time. Integrating constructs newly emergent from the 1970s, Crawford created a novel synthesis. His paper, like many other sociological analyses of the period, offered a challenge to medical dominance. However, unlike the medicalisation thesis that offered strident criticisms of medicine and its actions, Crawford argued that the solution was not simply to take back control. That strategy fell into an even bigger trap and furthered a political agenda that pursued the virtues of individual agency as the foundations of an emerging world. For Crawford, healthism ‘functions as dominant ideology, contributing to the protection of the social order from the examination, critique, and restructuring which would threaten those who benefit from the malaise, misery, and deaths of others’ (p. 369).

## 4 After 1980: Continuing the Discourse

Crawford's paper of 1980, as noted, accumulated over 2000 citations, of which just under 120 could be described as ‘influential’ as indicated by their own high citation counts. The vast majority of these highly cited publications endorsed Crawford's observation that healthism was spreading and, like Crawford, was critical of this phenomenon. In these citations, there was very little conceptual development of healthism: Most citations simply mentioned it, almost in passing. Understanding the impact of the idea of healthism in subsequent decades therefore needs an examination of the context in which it was used.

A few citations, especially in the early years, mentioned healthism but were more concerned with developing Crawford's observations on holism and self‐help/care as being too individualistic. Holistic health practice, for example, was seen to ‘place increasing areas of everyday life within the medical realm’ such that concepts such as stress, grief and friendship became linked in people's minds with health and illness, a sort of ‘medicalisation of lifestyle’ (Lowenberg and Davis 1994). Moreover, by situating the individual at the heart of health problems, holism opened them to corporate and entrepreneurial marketing efforts (Richards 1991). There was also agreement with Crawford that although alternative medicine seemed to ‘demedicalise’ personal health by encouraging the individual to be less dependent on biomedicine, it actually ‘remedicalises life’, bringing all areas of a person's emotional and spiritual life under further scrutiny (Cant and Sharma 1999, 44).

Most citations, however, used the concept of healthism, that is, ‘the population's growing occupation and fascination with health’ (Fýlkesnes and Førde 1992), in support of other arguments. Mention of healthism therefore provided a shorthand reference for an agreed common feature of Western societies. Only one citation offered empirical support for the thesis, noting that a Gallup poll found 84% of Americans mentioned good health as among the values they prized the most, confirming ‘what observers here and abroad view as our national preoccupation with health’ (Brandt and Rozin 1997).

According to Crawford, healthism was underpinned by the supreme value of ‘super health’, which subsumed various beliefs and mental states that were part of a certain aspirational lifestyle. However, the operational focus for achieving that goal was behaviour: ‘In healthism, healthy behaviour has become the paradigm for good living’ (p. 380). However, what were these healthy behaviours that underpinned healthism? In contrast to the wide range of states and activities embraced by medicalisation (Tiefer 1996), the terrain of healthism was much more circumscribed. Crawford mentioned smoking, alcohol consumption, diet and exercise (although noting the actual difficulty of changing these ‘lifestyles’). Although many citations simply echoed the idea that ‘the pursuit of good health has become an end in itself’ (Lupton 1995), the citations that applied healthism more specifically were often focused on exercise and diet; none addressed or was critical of individuals taking some responsibility for reducing tobacco or alcohol consumption.

Given the focus on exercise as a key behaviour, sociological studies of sport and physical education found healthism a useful explanatory framework (Bloyce and Smith 2010; Tinning 2010; Waddington and Smith 2000): ‘health‐based physical education programmes, which project and celebrate corporeal and individualistic notions of health, take on enormous significance since they explicitly evoke the moral order’ (Kirk and Colquhoun 1989). A boom in recreational running in many affluent countries of the Global North, in aerobics and the like, was an expression of a growing population‐wide perception of the need to exercise on a regular basis to combat the ‘mal‐effects of sedentary behaviour’: physical education attempted to prevent those individuals ‘upstream’ and on the ‘riverbank of life’ from becoming ill (Kirk 2019). Similarly, sociological studies of diet found healthism a useful construct in explaining the rise of the nutricentric person (Scrinis 2013). Indeed, healthism meant the simple acts of cooking and eating needed constant attention by those who became their own ‘food practitioners’ (Halkier 2010).

Crawford had, as noted, acknowledged the benefits for health of pursuing these behaviours: ‘My critique of healthism is not aimed at questioning whether it has any therapeutic value. Anything that works for the individual cannot be dismissed’ (p. 385). However, for Crawford, the costs of healthism outweighed the benefits. First, healthism was an ideology largely endorsed by the middle class (who were in a position to follow its precepts), whereas more disadvantaged members of the community had other priorities; it therefore increased health inequalities. Second, it resulted in stigma and victim‐blaming for those unable or unwilling to embrace the new healthism ideology. And thirdly, by focusing on individual responsibility, healthism distracted attention from the social and environmental factors that were also implicated in good health.

All these costs found echo in subsequent citations. Advocates of a healthy lifestyle appeared to be ‘overwhelmingly middle class’ (Black 2004); healthism bore a ‘middle‐class stamp’ (Monaghan 2008); healthism perpetuated a ‘medicalized middle class’ (De Leeuw 2017). Further, because embracing healthism was a moral duty, those who failed to do so exposed themselves to moral disapproval (Howarth 2007). Subsequent citations also agreed that healthism distracted from social and environmental factors in maintaining health. ‘The whirlwind of risks’ (of cholesterol levels, of coffee, salt, sugar etc.) ‘depoliticises the wider causes of sickness and disease and leaves us blaming ourselves for conditions that are outside our control’ (White 2002). Or, as Helman described it, ‘other factors ‐ such as poverty, unemployment, economic crises, pollution or persecution—(are) often ignored by the medical system because its main focus is increasingly on the individual patient and the “risk factors” in his or her own lifestyle’ (Helman 1990, 66).

Crawford had agreed that an individualistic focus would distract from wider causes of illness and disease but seemed ambivalent about some of the alternatives. To be sure, healthism took attention away from more social and environmental solutions, but he was also sceptical of the alternative occupational and environmental health movements for their focus on factors external to the individual. He thought that ‘The ideologically imposed and disabling separation between private, personal, coping actions and political movements designed to change society must be overcome if a viable health strategy and, indeed, a viable society are to be achieved’ (p. 385). In other words, Crawford suggested that some sort of balance was needed between those political activists focused on factors external to the individual and healthists (in his case in the holistic health and self‐care movements) who were preoccupied with the subjective, behavioural arena: ‘Both take fundamental truths and turn them into half‐truths through an exclusive attention. One takes the individual as the problem; the other takes the society as the problem’ (p. 384).

Citations to healthism, however, were more focused on the costs and not on any benefits, nor on any balancing calculation. That task was left to others. It was not fellow sociologists who provided the alternative arguments but medical authors and governments. On the one hand, the vision of patients not taking some responsibility for their health invoked the ‘nanny state’, which libertarians opposed (Fitzpatrick 2001; Le Fanu 1999). On the other hand, governments were concerned about the involvement of patients in their health to offset the rising costs of health care for the public purse. Here, the expectation that citizens play a more active role in caring for themselves was expressed in campaigns to ‘look after yourself’ and in analyses that showed in future years state funding of health care (in the UK) would be impossible if citizens were not ‘fully engaged’ in their own health and health care (Wanless 2002). In other words, the benefits of healthism accrued not only to individuals but also by reducing demand on existing and future services, to others.

Overall, the discourse of healthism that was sustained for several decades by sociologists was concerned with maintaining the argument that healthism actually posed threats to health in the round. The dominance of the medical discourse promoting some degree of self‐reliance and self‐responsibility for health perhaps needed rebalancing with a more critical perspective, and citations to healthism provided that alternative view. Healthism also provided a reference point for other analyses of modern society.

## 5 Body Beautiful

In 1987, Scheper‐Hughes and Lock published an influential study (with over 4000 citations, the highest‐impact publication referencing Crawford's paper) on the importance of the body for medical anthropology (Scheper‐Hughes and Lock 1987). In this article, they proposed the ‘deconstruction of received concepts about the body’. They noted that ‘in our own increasingly “healthist” and body‐conscious culture, the politically correct body for both sexes is the lean, strong, androgenous, and physically “fit” form … each individual is expected to “work hard” at being strong, fit, and healthy’ (p. 25). These, indeed, were the core elements of healthism but presented in this account with a particular focus on the body, and over the following decades, the body became both the substrate on which healthism worked and the manifestation of its success. For Crawford, the body was simply either one side of the ‘mind‐body’ duality or the container for disease. Although Crawford never explicitly mentioned ‘appearance’ in his paper, in the decades that followed, increasing focus on the body, its shape and its size came to dominate much of the healthism literature.

There were broader contemporary shifts that accentuated attention to the appearance of the body. For most of the preceding century, exposing the body had been surrounded by social taboos and restrictions, but in the closing decades of the 20^th^ century, the body became something to be displayed. And the body became a key element in the new health consciousness ‘because it is in and through body shape, size and its capacities to achieve physical tasks that health is measured’ (Kirk and Colquhoun 1989). ‘Health, fitness, youthfulness and beauty came together in a particular moral form’ (Petersen and Bunton 1997). A prime reason for taking exercise and eating healthily was in order to keep ‘in shape’ (Kirk 2019). ‘Lookism’ became a key bedfellow of healthism (Ayalon and Tesch‐Römer 2018); a focus on body size and shape became the new ambition (Håman et al. 2015). In effect, the pursuit of good health was not the only motivation for doing exercise and carefully controlling diet, but also weight, size and shape. Concern with appearance informed analysis of phenomena such as tattooing, piercing and body dysmorphia (Negrin 2008). Other strategies, such as nutritional supplements and surgical procedures, often as part of ‘medical tourism’ (Lunt et al. 2010), could then be added to aerobics and gym membership (Andreasson and Johansson 2014; Brighton et al. 2021) in the attempt to attain a public face of good health.

The confluence of healthism and appearance found its perfect expression in citations that addressed obesity. Obesity implicated diet and exercise—two central behaviours underpinning healthism. ‘An additional layer of morality has been added to body weight and eating as controlled appetite and trim bodies have come to represent healthy living in a society where the pursuit of health is a moral end in itself’ (Saguy and Almeling 2008, 55). The concept of healthism explained how ‘an attractive and youthful body [is] an element and marker of good health [whereas] an obese body represents laziness, emotional weakness, and unattractiveness’ (Håman et al. 2015, 2). A study of weight gain in women reported that many older women expressed a sense of guilt and shame for their inability to adhere to societal understandings of healthy living by disciplining and controlling their weights and thereby rendering their bodies attractive as well as healthy. Thus, it was concluded, women's appearance evaluations were strongly influenced by healthist discourses that held individuals personally responsible for the health and appearance—‘which are assumed to be one and the same thing’—of their bodies (Clarke 2011).

In the main, those authors contributing to the discourse on healthism and obesity deprecated the stigma the latter attracted. ‘Health (or their perceived lack of health) is used as a weapon against fat people in their fight for civil rights. On the front lines of health and the “war on obesity” are healthcare providers. Anti‐fat attitudes in healthcare providers are incredibly well documented (Lee and Pausé 2016, 5): ‘today there is a pervasive and hostile anti‐fat ethic’ (Monaghan 2008). In the main, these opponents of ‘fatism’ did not therefore explore or balance the potential health costs from obesity (though there were exceptions [Saguy and Riley 2005]); those were left to medicine to publicise. In their study on the role of the body in future medical anthropology scholarship, Scheper‐Hughes and Lock speculated that if illness was in some way attributed to the individual’s failure to live right, to eat well and to exercise, then a key question was what it is our society ‘wants’ from this kind of body? (Scheper‐Hughes and Lock 1987). Medicine provided one answer; sociology, through healthism, a critical alternative.

## 6 The Changing Boundaries of Responsibility

Healthism represented a turn inward towards self‐cultivation, Crawford suggested, itself a reaction to the disillusionment and disappointment of the 1970s after the political excitement of the 1960s. Later authors, trying to explain the rise of healthism, identified it as an offshoot of conservative ideology, rooted in the belief that people reap what they sow (Cheek 2008). For others, healthism was both like a religion and a form of oppression in that what could no longer be done in the name of morality could now be done in the name of health (Lee and Pausé 2016), or healthism was a strategy to toughen the population in preparedness for war. Indeed, the self‐help and fitness movements articulated both a militarist and a Social Darwinist ethos: ‘the fast and fit win; the fat and flabby lose and drop out of the human race’ (Scheper‐Hughes and Lock 1987, 25).

The political consequences of healthism were also debated. A minority view was that healthism resulted in heightened individual political consciousness, a precondition for social action (Katz and Levin 1980). Crawford, though, had been sceptical: ‘individual responsibility as ideology has often functioned historically as a substitute for collective political commitments’ (p. 378). The linking of health, morality and blame that characterised healthism undermined the no‐fault principle in the classical sick role (Green et al. 2015). In a context where health consciousness had become increasingly unavoidable, healthy subjects had to seek out, assess and act upon an endless stream of knowledge on the latest health threats—an information‐saturated environment that fostered anxieties about seemingly pervasive dangers (Cairns and Johnston 2015).

Healthism underpinned claims that citizens were increasingly expected to play a more active role in caring for themselves—a shift from the mid‐century understanding that the state had primary responsibility for the health and welfare of its citizens (Sharon 2017). In fact, in the decades after 1980, healthism became a marker of the transformation of the relationship between individuals and government in a wider shift from welfare to what were described as neoliberal models of governance in advanced Western societies (Kirk 2019; Sloan et al. 2010; White 2002). For many authors, a healthist society came to be described as and equated with a neoliberal society. Crawford himself came to accept that healthism had become ‘a model of and a model for the neoliberal restructuring of American society’ (Crawford 2006). Though some analysts suggested that rather than the state taking a step back, ironically, healthism allowed the state to enter more into people's lives: ‘somewhat ominously … some of the worst totalitarian states in history have been enthusiastic supporters of “healthism”’ (Baggott 2011, 10).

The epidemiological shaping of risk was also part of neoliberal society: Risks were constructed as individual events people were responsible for, though, it was observed, many were not amenable to intervention at the individual level (White 2002). Healthism also provided a point of articulation for a growing concern and analysis of the social determinants of health. For those not in a position to ‘choose health’ and for those risks that were beyond individual actions (such as pesticides in food, carcinogens in the air, capacity to bus or bike instead of driving), the construct of healthism ‘opened a space for thinking about health as contingent, multivalent, and complexly intertwined with our social and material environments’ (Shotwell 2016). As Evans noted, a strategy seeking both to blame and empower the individual was ‘highly political’ (Evans et al. 2004). Whatever the empirical costs and benefits of healthism, its significance also lies in the ways it came to be used to reflect and shape contemporary political positions.

Although, for Crawford, medicalisation and healthism were separate if parallel strategies, in subsequent publications, their relationship became a topic for debate. Conrad, for example, suggested that healthism would be better conceptualised as ‘healthicization’: ‘With medicalisation, medical definitions and treatments are offered for previous social problems or natural events; with healthicization, behavioural and social definitions are advanced for previously biomedically defined events (e.g. heart disease)’. On the one hand, medicalisation proposed biomedical causes and interventions; on the other hand, healthicization proposed lifestyle and behavioural causes and interventions. ‘One turns the moral into the medical, the other turns health into the moral’ (Conrad 1992, 223). De Leeuw argued that the health care system, as shaped through the medicalisation of everyday life events, enforcing a medicalised middle class, was gradually extending medicalisation insidiousness into health rather than disease (De Leeuw 2017). Indeed, the proliferation of medical and epidemiological studies that started in the 3 decades before healthism was described have promoted the pursuit of health through the very activities—such as a healthy diet and appropriate exercise—that have underpinned the healthism thesis.

Some revision of medicalisation was also brought about by the increasing involvement of patients in their own health care alongside medicine. Healthism was presented as a ‘lay’ pursuit in contrast with the activities of doctors. However, healthism can also be regarded as an extension of the practice of medicine into the population. In other words, medical care was no longer restricted to doctors but increasingly extended also to patients: Patients were becoming their own lay practitioners. The emergence of the ‘expert patient’, often promoted by sociology (Tuckett et al. 1985), recognised that patients can be specialists in their own, often chronic, diseases. It was the patient who needed to manage and monitor their own care (e.g., in diabetes) if optimal outcomes were to be achieved. Healthism was therefore a form of medicalisation in the sense that patients had become practitioners. In 1980 a key consequence of medicalising behaviour was to diminish or remove blame from the individual (Conrad and Schneider 1980)—the opposite of healthism. In subsequent years, it has become less clear where responsibility lay when patients were encouraged to manage their chronic illnesses in partnership with medical practitioners or when there was ‘shared decision‐making’ in an enlightened clinical consultation.

## 7 A Changing Subject of Knowledge

The core underpinnings for the idea of healthism in 1980 were some significant constructs that had only emerged during the previous decade. These included medical dominance/power, medicalisation, alternative medicines, lifestyles, self‐responsibility and health behaviours. Healthism provided a useful reference point for publications addressing these themes in some way. However, in the most recent decades, other constructs such as neoliberalism and appearance/obesity have provided important new contexts for positioning the concept and thereby extended its ‘influence’. To be sure, self‐responsibility remains a core component of analyses of healthism. However, although formerly discussion was framed in terms of the role of responsibility in the Parsonian sick role, latterly interest has been more in the changing interplay between citizen and state, as expressed in debates around neoliberalism. In that conceptual space between individual citizen and state, healthy behaviours and self‐responsibility could be recast. Indeed, self‐responsibility and neoliberalism both started to rise in popularity (according to Google Ngrams) at exactly the same time as citations to healthism started to increase. Similarly, the emergence of other discourses, particularly around body appearance, during the early years of the new millennium provided a new source of citations to healthism. Reference to health behaviours around diet and exercise, for example, became so commonplace that the term ‘healthism’ provided a useful placeholder for a widely understood lifestyle. Any author wishing to describe the preoccupation with healthy behaviours had only to reference the 1980 paper.

As Crawford recognised, referencing Foucault, the medical model, in which illnesses were the result of lesions inside the body, defined the limits for which solutions for health problems were sought. For Crawford: ‘The individual is both the locus of perception and intervention, more firmly so since … the very foundation of medical knowledge becomes lodged in the “sovereignty of the gaze” fixed on individual signs and symptoms and then in deep anatomical structure’ (p. 371). Despite their drawbacks, at least holism, self‐help/care and healthism located the problem in the wider context of ‘lifestyle’. A lifestyle approach also helped differentiate medicalisation with its reductionist gaze from healthism with its outside‐body‐boundary focus, especially on behaviours involving diet and exercise.

However, according to Foucault, that historical shift two centuries ago, when illness became a consequence of a pathological lesion, was also a moment when it became possible that ‘Western man could constitute himself in his own eyes as an object of science’ (Foucault 1973, 197). In other words, the medical revolution that located illness to an intracorporal lesion and that introduced intensive scrutiny of individual bodies through new techniques such as the clinical examination and autopsy/post‐mortem established an objectified body that, in turn, could be further studied. This circumscribed body, this new ‘object of science’, underpinned an emerging sense of individualism. At once, the individual became ‘both subject and object of his own knowledge’ (p. 197). Pathological medicine might have constrained views of how best to achieve good health, but it was also foundational in the very idea of the individual.

In a similar vein, the ‘new’ clinical approaches, whether in the role of extra‐corporal risk factors or the self‐practitioner element underpinning healthism, might be seen as a continuation of that process, in this case of an emerging subjectivity as reflected in ideas about self‐responsibility. Turrini (2015) argued for the importance of healthism in its relation to autonomous individualism, self‐control, self‐determination and self‐responsibility: ‘Health has become a vector for the production of the self and the formation of neoliberal subjectivities that introduces the faculty of choice and free will into the everyday management of our body through risk assessment’ (Turrini 2015, 22). From risk factors through neoliberalism to bodily appearance, the alliances that healthism has forged furthered this aim. In this analysis, healthism is a component of subjectification, part of an ongoing process that started two centuries ago but ‘has not yet been unravelled’ (Foucault 1973, 220). The influence of healthism over the decades lies in the role it has played in shaping and promoting wider discourses, from lifestyles and health behaviours to neoliberalism and appearance, but its enduring influence might be expressed in the role it has played, alongside these other analyses, in the construction of the modern subject.

## Author Contributions

**David Armstrong:** conceptualization (lead), methodology (lead), formal analysis (lead), writing – original draft (lead), writing – review and editing (lead).

## Ethics Statement

The author has nothing to report.

## Conflicts of Interest

The author declares no conflicts of interest.

## Data Availability

Data for this paper are derived from publicly available publications.
